# Protein Requirements Are Elevated in Endurance Athletes after Exercise as Determined by the Indicator Amino Acid Oxidation Method

**DOI:** 10.1371/journal.pone.0157406

**Published:** 2016-06-20

**Authors:** Hiroyuki Kato, Katsuya Suzuki, Makoto Bannai, Daniel R. Moore

**Affiliations:** 1 Frontier Research Laboratories, Institute for Innovation, Ajinomoto Co., Inc., Kawasaki, Kanagawa, Japan; 2 Faculty of Kinesiology and Physical Education, University of Toronto, Toronto, Ontario, Canada; University of Alabama at Birmingham, UNITED STATES

## Abstract

A higher protein intake has been recommended for endurance athletes compared with healthy non-exercising individuals based primarily on nitrogen balance methodology. The aim of this study was to determine the estimated average protein requirement and recommended protein intake in endurance athletes during an acute 3-d controlled training period using the indicator amino acid oxidation method. After 2-d of controlled diet (1.4 g protein/kg/d) and training (10 and 5km/d, respectively), six male endurance-trained adults (28±4 y of age; Body weight, 64.5±10.0 kg; VO_2_peak, 60.3±6.7 ml·kg^-1^·min^-1^; means±SD) performed an acute bout of endurance exercise (20 km treadmill run) prior to consuming test diets providing variable amounts of protein (0.2–2.8 g·kg^-1^·d^-1^) and sufficient energy. Protein was provided as a crystalline amino acid mixture based on the composition of egg protein with [1-^13^C]phenylalanine provided to determine whole body phenylalanine flux, ^13^CO_2_ excretion, and phenylalanine oxidation. The estimated average protein requirement was determined as the breakpoint after biphasic linear regression analysis with a recommended protein intake defined as the upper 95% confidence interval. Phenylalanine flux (68.8±8.5 μmol·kg^-1^·h^-1^) was not affected by protein intake. ^13^CO_2_ excretion displayed a robust bi-phase linear relationship (R^2^ = 0.86) that resulted in an estimated average requirement and a recommended protein intake of 1.65 and 1.83 g protein·kg^-1^·d^-1^, respectively, which was similar to values based on phenylalanine oxidation (1.53 and 1.70 g·kg^-1^·d^-1^, respectively). We report a recommended protein intake that is greater than the RDA (0.8 g·kg^-1^·d^-1^) and current recommendations for endurance athletes (1.2–1.4 g·kg^-1^·d^-1^). Our results suggest that the metabolic demand for protein in endurance-trained adults on a higher volume training day is greater than their sedentary peers and current recommendations for athletes based primarily on nitrogen balance methodology.

***Trial Registration***: ClinicalTrial.gov NCT02478801

## Introduction

It has been recommended that highly active and trained individuals should consume protein intakes greater than the current recommended daily allowance (RDA; 0.8 g/kg/d), the latter of which was developed in healthy non-exercising populations. For instance, the protein intake for endurance-trained athletes is recommended to be 1.2–1.4 g protein ·kg^-1^·d^-1^ [1], which is reflected in many sports science consensus statements [1–3] and may be related in part to the associated increase in amino acid oxidation during endurance exercise [4]. The protein requirements and recommended protein intake in endurance-trained individuals have been investigated primarily through the nitrogen balance (NBAL) technique. However, this method has been suggested to have limitations (e.g. see [5]) including a general predisposition to overestimate nitrogen intake and underestimate nitrogen excretion [6], which collectively would result in an underestimation of true protein requirements [7]. Given the importance of dietary protein for the repair and remodeling of body proteins in active populations, there is a need to re-evaluate current protein recommendations for endurance athletes with alternative methodologies to provide collective evidence as to the true protein requirements in this population.

The minimally-invasive indicator amino acid oxidation (IAAO) method was developed as an alternative to the traditional NBAL technique as a means to assess the individual amino acid and protein recommendations in a variety of populations [8, 9]. The limited dietary adaptation period required for the IAAO method relative to NBAL technique (e.g. 1 vs. 5–7 days, respectively) allows for a greater number of test protein intakes to be performed within a given participant [10, 11]; this allows for bi-phase modeling of the data that has been suggested to be more robust compared with linear modeling [7]. As such, the IAAO method has been applied in a variety of human studies to determine the recommended intake of protein and individual amino acid [7, 12–14]. However, the IAAO method has yet to be applied in active individuals, let alone endurance-trained athletes. Therefore, the aim of the present study was to apply the IAAO method to determine the estimated average protein requirement and recommended protein intakes in endurance trained individuals. We hypothesized that the IAAO method would result in a recommended protein intake in our endurance-trained population that would be greater than the current RDA of 0.8 g·kg^-1^·d^-1^ as well as the recommended protein intake of 1.2–1.4 g·kg^-1^·d^-1^ in similarly trained athletes [1] as determined by NBAL.

## Methods

### Ethics Statement

**Fig 1:**
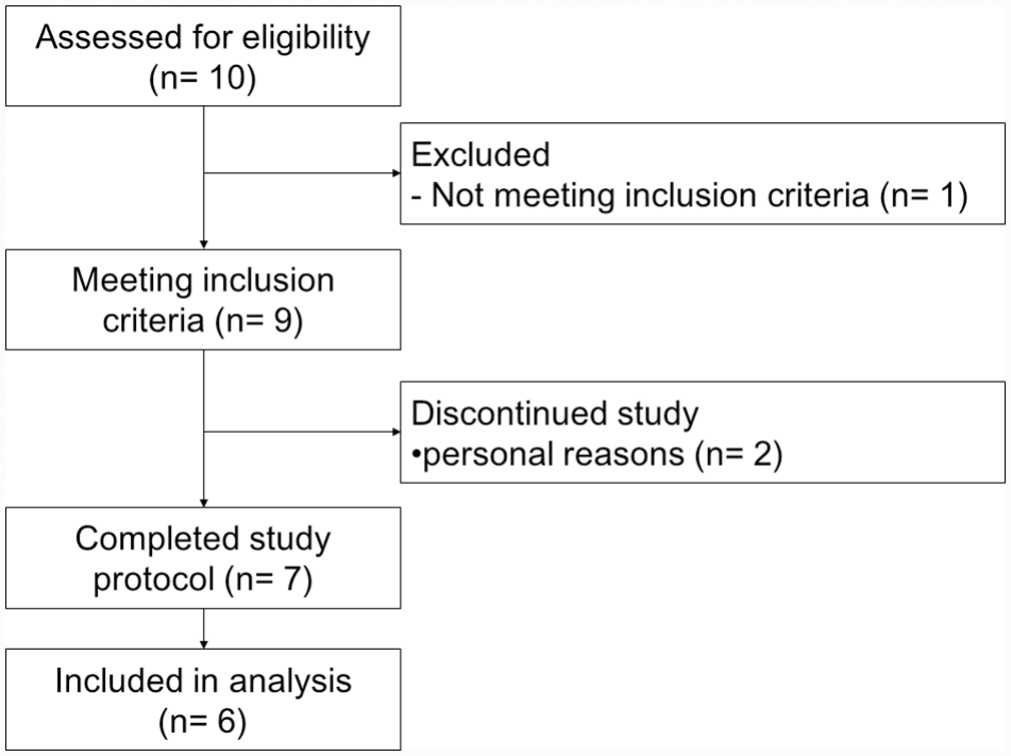
Flowchart of the trials.

All participants were informed of the purpose of the study, the experimental procedures, and all the potential risks involved before obtaining written consent. This study was conducted in accordance with the Declaration of Helsinki, and the protocol was approved by the research ethics board of the University of Toronto on 17^th^ March, 2015 and the institutional review board of Ajinomoto Co., Inc. on 24^th^ December 2014. Informed written consent was obtained from all the participants. This trial was registered at clinicaltrial.gov as NCT02478801 after the recruitment had begun due to an unfortunate oversight. The authors confirm that all ongoing and related trials for this intervention are registered. The participants were recruited from 24^th^ March 2015 to 5^th^ Jun. 2015. The study was conducted from 25^th^ March. 2015 to 25^th^ Jul. 2015. A detailed flow of the trials is described in Fig 1. The protocol for this trial and a CONSORT checklist are available as (S1 CONSORT Checklist and S1 Protocol).

### Study protocol

Before beginning the studies, each participant visited the Goldring Centre for high performance sport after an overnight fast (~7 h) to have their body composition [Fat mass (FM) and Fat-Free Mass (FFM)] determined by Bodpod (Cosmed USA Inc., Chicago, IL). Following the body composition, participants sat comfortably in a darkened room to determine their resting energy expenditure (REE) for 25 min by continuous, open-circuit indirect calorimetry (MOXUS metabolic cart; AEI technologies Inc., Bastrop, TX) and application of the abbreviated Weir equation [15]. Participant’s aerobic fitness was assessed by measurement of respiratory gas exchange throughout a ramp protocol exercise test to determine their maximal oxygen consumption (VO_2peak_), as previously described [16]. Briefly, participants began running on a treadmill a light pace after which the work rate increased at a constant, linearly rate. The test was completed in ~12min after participants reached a point in which they could no longer continue (volitional fatigue).

Each randomly assigned level of protein was studied over a 3-d period, which included 2 adaptation days followed by a metabolic trial day. During the 2 adaptation days, participants performed pre-set standardized exercise, which involved a 10-km run on the first day and a 5-km run on the second day at a self-selected running pace to ensure each participant performed similar physical activity prior to the metabolic trial day. The combination of the 2-d controlled training with the trial day exercise stimulus of 20 km (see below) resulted in a total training volume of 35 km over 3 days, which was within the general habitual training volume of the participants.(i.e. self-reported at ~45–130 km/wk). During the 2 adaptation days, participants consumed adaptation diets that included commercially available, pre-packed or frozen foods. The energy content of the controlled diet was estimated as 1.6 times REE plus the exercise-induced energy expenditure (EEE) estimated from the pre-set standardized exercise as 1 kcal·kg^-1^·km^-1^ [17]; this energy expenditure estimate was selected to be sufficient to offset the actual energy cost of the exercise given that our participants performed the exercise on a level treadmill (see below) that is metabolically more efficient than running on a road [18]. The adaptation diet supplied a moderate 1.4 g protein·kg^-1^·d^-1^ in accordance with the current recommended protein intake for endurance-trained athletes [1] in order to standardize the protein intake and minimize metabolic variability on the trial day [19]. The adaptation diet also provided 8.0 g carbohydrate·kg^-1^·d^-1^, which is consistent with current consensus recommendations for endurance athletes training 1–3 h·d^-1^ [20]. On the 3^rd^ day, after overnight fasting (~7 h), participants completed the metabolic trial (see below for details). Each trial was separated by at least 4 days with all trials for a given participant completed within 4 months.

### Metabolic trial

The metabolic trial protocol was similar to those previously used to estimate protein requirements in non-exercising populations [7, 12] with the exception that the present study included an exercise stimulus. On the metabolic trial day, participants arrived at the laboratory after an overnight fast and consumed a protein-free liquid carbohydrate beverage [1.2 g carbohydrate·kg^-1^·d^-1^ as a 1:1 ratio of maltodextrin (Polycal^®^; Nutricia, Amsterdam, Netherlands) and sports drink powder (Gatorade^®^ Endurance Formula; PepsiCo, Purchase, NY)] 1h prior to the exercise to help to replenish liver glycogen and provide some exogenous carbohydrate energy to fuel the 20-km run [20]. Participants then completed a 20-km run at a self-selected race pace on a motorized treadmill while wearing a heart rate (HR) monitor sensor strap (Polar Electro, Kempele, Finland) on their chest in order to estimate the exercise-induced energy expenditure according to Crouter’s equation [21]. During the exercise participants were provided with their accumulated mileage, HR, and running pace.

Immediately following exercise, participants received the study diet containing a randomly assigned protein intake (0.20 ~ 2.8 g protein ·kg^-1^·d^-1^; Table 1) as 8 isocaloric and isonitrogenous hourly meals that each provided one-twelfth of the participant’s total daily energy requirement. The study diet was provided in the form of protein-free cookies [22] and test drinks, the latter of which contained protein-free powder (PFD-1; Mead Johnson, Evansville, IN), flavoring crystals (Tang; Kraft, Don Mills, Canada), grape seed oil, maltodextrin (Polycal^®^), and a crystalline amino acid mixture (Ajinomoto North America, Inc., Raleigh, NC). The amino acid pattern of the test protein intake was modeled on the basis of egg protein (Table 2) with the exception of phenylalanine and tyrosine, which were held constant at an intake of 30.5 and 40.0 mg·kg^-1^·d^-1^, respectively. The inclusion of excess tyrosine is to ensure metabolic partitioning of the carboxyl carbon of phenylalanine towards protein synthesis or oxidation during stable isotope ingestion [23, 24]. The study diet and the protein-free liquid carbohydrate beverage provided sufficient energy (i.e. 1.6 * REE plus EEE estimated from 20-km run as 1 kcal·kg^-1^·km^-1^ [17]) and carbohydrate, the latter of which, when combined with the pre-exercise beverage, would result in ~9.0 g·kg^-1^ carbohydrate·d^-1^. As such, by providing sufficient energy intake during the IAAO study we would ultimately minimize amino acid oxidation and subsequently determine a minimum protein intake in our population. A priming dose of NaH^13^CO_3_ (0.176 mg·kg^-1^; CIL Canada, Inc., Montreal, Canada) and L-[1-^13^C]phenylalanine (1.86 mg·kg^-1^; CIL Canada, Inc., Montreal, Canada) was ingested in the 5^th^ test drink [7, 13, 14]. All subsequent test drinks during the metabolic trial included 1.20 mg·kg^-1^ of L-[1-^13^C]phenylalanine as part of the total intake to maintain isotopic steady state until the end of the metabolic trials.

**Table 1:** Protein intakes used in individual subjects. Participants consumed each protein intake which ranged from 0.2 to 2.8 g·kg^-1^·d^-1^, for a total of 34 trials.

Subject No.	Test protein intakes, g·kg^-1^·d^-1^
1	0.2, 0.9, 1.3, 1.65, 1.8, 2.3, 2.8
2	0.8, 1.15
3	0.4, 1.0, 1.6, 2.0, 2.65
4	0.45, 0.7, 1.05, 1.45, 1.7, 2.35, 2.5
5	0.5, 0.6, 1.4, 1.95, 2.25, 2.6
6	0.25, 0.85, 1.2, 1.5, 1.75, 2.15, 2.75

**Table 2:** Amino acid composition of reference protein and selected test protein intakes^1^.

	Reference protein^2^, mg·g^-1^"	0.2 g protein·kg^-1^·d^-1^	0.7 g protein·kg^-1^·d^-1^	1.2 g protein·kg^-1^·d^-1^	1.7 g protein·kg^-1^·d^-1^	2.2 g protein·kg^-1^·d^-1^	2.8 g protein·kg^-1^·d^-1^
L-Alanine	61.5	12.3	43.1	73.8	104.6	135.3	172.2
L-arginine HCL^3^	75.1	15.0	52.6	90.1	127.7	165.2	210.3
L-Asparagine	33.3	6.7	23.3	40.0	56.6	73.3	93.2
L-Aspartic acid	33.3	6.7	23.3	40.0	56.6	73.3	93.2
L-Cysteine	22.1	4.4	15.5	26.5	37.6	48.6	61.9
L-Glutamine	56.6	11.3	39.6	67.9	96.2	124.5	158.5
L-Glutamic acid	56.6	11.3	39.6	67.9	96.2	124.5	158.5
L-Glycine	33.3	6.7	23.3	40.0	56.6	73.3	93.2
L-Histidine	22.7	4.5	15.9	27.2	38.6	49.9	63.6
L-Isoleucine	62.8	12.6	44.0	75.4	106.8	138.2	175.8
L-leucine	83.3	16.7	58.3	100.0	141.6	183.3	233.2
L-Lysine HCL^2^	75.7	15.1	53.0	90.8	128.7	166.5	212.0
L-Methionine	29.6	5.9	20.7	35.5	50.3	65.1	82.9
L-Phenylalanie^3^	54.7	30.5	30.5	30.5	30.5	30.5	30.5
L-Proline	41.9	8.4	29.3	50.3	71.2	92.2	117.3
L-serine	83.9	16.8	58.7	100.7	142.6	184.6	234.9
L-threonine	47.1	9.4	33.0	56.5	80.1	103.6	131.9
L-tryptophan^4^	15.6	3.1	10.9	18.7	26.5	34.3	43.7
L-Tyrsoine^5^	40.7	40.0	40.0	40.0	40.0	40.0	40.0
L-Valine	70.3	14.1	49.2	84.4	119.5	154.7	196.8

^1^ Participants consumed a single protein intake that ranged from 0.2 to 2.8 g·kg^-1^·min^-1^, on each metabolic trial.

^2^ Represents egg protein composition.

^3^ Actual concentration of amino acid in HCl form in amino acid mixture; arginine, 62.1 mg·g^-1^; and lysine 60.6 mg·g^-1^.

^4^ Phenylalanine intake was held constant at 30.5 mg·kg^-1^·d^-1^ for all protein intakes.

^5^ Tyrosine intake was held constant at 40.0 mg·kg^-1^·d^-1^ for all protein intakes

### Sample collection and analysis

Three baseline breath samples (45, 30, and 15 min) and 2 baseline urine samples were (45 and 15 min) before the participants consumed the 5^th^ test drink containing the indicator amino acid. Six plateau breath samples were collected every 15 min and three plateau urine samples were collected every 30 min beginning 2.5h after the 5^th^ test drink. Steady state CO_2_ production (VCO_2_) was measured for 20 min ~30 min after the 5^th^ or 6^th^ test drink by indirect calorimetry (MetaMax 3B, CORTEX Biophysik GmbH, Leipzig, Germany). These time points of breath sampling were selected according to pilot testing that confirmed background ^13^CO_2_ enrichment from the diet (in the absence of tracer ingestion) and VCO_2_ were both constant, indicating stable background isotopic and metabolic steady state was achieved (S1 Text). Breath samples were collected in disposable Extainer tubes (Labco, Ltd., Ceredigion, UK) with a collection system (Easy-Sampler; QuinTron Instrument Company, Inc., Milwaukee, WI) that permitted the removal of dead-space air. Breath samples were stored at room temperature prior to measurement of ^13^CO_2_ enrichment by a continuous-flow isotope ratio mass spectrometry (CF-IRMS 20/20 isotope analyzer; PDZ Europa Ltd, Cheshire, UK). Urine samples were stored at -20°C prior to [1-^13^C]phenylalanine enrichment determined by API 4000 triple quadrupole mass spectrometer (Applied Biosystems, Foster City, CA) in positive electrospray ionization mode, as previously described [12, 25].

### Tracer kinetics

Phenylalanine flux (PheRa, μmol·kg^-1^·h^-1^), the rate of appearance of ^13^CO_2_ in breath (F^13^CO_2_; μmol·kg^-1^·h^-1^), and phenylalanine oxidation (PheOx; μmol·kg^-1^·h^-1^) were calculated according to the stochastic model of Matthews et. al.[26] as follows:

$$\text{PheRa}=\text{i }\cdot \left(\frac{E_{i}}{E_{u}}\right)-I$$

Where i is the rate of L-[1-^13^C] phenylalanine ingested (μmol·kg^-1^·h^-1^), I is the rate of L-phenylalanine ingested (μmol·kg^-1^·h^-1^), E_i_ and E_u_ are the isotopic enrichments as mole fractions (APE) of the test drink and urinary phenylalanine, respectively, at isotopic plateau.

$$F^{13}CO_{2}\;=\;\left(V_{CO_{2}}\right)\cdot \left(E_{CO_{2}}\right)\cdot \left(44.6\right)\cdot \left(60\right)\cdot BW^{-1}\cdot \left(0.82\right)\cdot \left(100\right)$$

Where VCO_2_ is the CO_2_ production rate (mL·min^-1^); E_CO2_ is the ^13^CO_2_ enrichment in expired breath at isotopic steady state (APE); BW is the body weight (kg) and FFM is the fat-free mass (kg) of the participants, as needed. The constants 44.6 (μmol·mL^-1^) and 60 (min·h^-1^) were used to convert F_CO2_ to μmol·h^-1^. The factor 0.82 is the correction for CO_2_ retained in the bicarbonate pool of the body in the fed state [27]. PheOx was calculated using Eu as an estimate of intracellular enrichment [28] as:

$$\text{PheOx }=\;F^{13}CO_{2}\cdot {\left(\frac{1}{Eu}-\frac{1}{Ei}\right)}^{-1}\times 100$$

### Statistical analysis

Unless indicated otherwise, all results are expressed as means ±standard deviation (SD). Protein or specific amino acid requirements determined by the IAAO method have previously been reported in non-exercising populations with 35–56 trials [7, 13, 25, 29]. Given that providing a range of test intakes provides a better modeling fit than 7 discrete intakes [7, 25] and that there is no difference between the goodness of fit between 35 and 43 trials using this approach (i.e. R^2^ = 0.60 and 0.63, respectively), we aimed to complete a target of n = 35 metabolic trials in the present study.

A mixed linear model with the subject as a random variable by using Proc Mixed program (SAS university version; SAS Institute Japan, Tokyo, Japan) was used to analyze the effects of protein intake on F^13^CO_2_, phenylalanine flux, and phenylalanine oxidation. A biphasic linear regression crossover analysis was performed on F^13^CO_2_ to determine the average protein requirement and recommended protein intakes in agreement with previous studies [7, 25, 30]. Protein intake at the breakpoint represented the average protein requirement, and recommended protein intake was estimated as the upper limit of 95% CI of the breakpoint. The 95% CI was calculated with use of Filler’s Theorem, as previously described [30].

## Results

### Participant’s characteristics

Nine participants who regularly run at least 40 km/week were recruited to participate. However, two participants discontinued due to personal reasons and one participant who only completed a single metabolic trial was excluded from data analysis. Therefore, the data from six participants were used for analysis with the participant characteristics summarized in Table 3 and the exercise stimulus summarized in Table 4.

**Table 3:** Characteristics of participants.

	Mean ± SD
Age, yr	28.3 ± 4.2
Height, cm	173.3 ± 4.0
Body weight, kg	64.5 ± 10.0
Fat-free mass, kg	56.5 ± 7.1
VO2peak, ml/kg/min	60.3 ± 6.7
REE, Kcal/day	1624.1 ± 274.3

**Table 4:** Summary of the endurance exercise stimulus in individual subjects. Values are means ± SD.

Subject No.	Duration (min)^1^	Intensity (%HR max)^2^	Exercise-induced energy expenditure (Kcal)^3^
1	96.1 ± 3.9	65.9 ± 3.5	878 ± 69
2	90.5 ± 4.9	71.3 ± 2.5	939 ± 112
3	90.8 ± 1.6	82.3 ± 3.3	1264 ± 80
4	91.4 ± 2.4	79.6 ± 1.7	1327 ± 68
5	102.2 ± 1.6	70.9 ± 3.8	1110 ± 109
6	118.3 ± 5.1	78.4 ± 2.4	1209 ± 50

^1^: Average time to complete 20 km on each metabolic trial.

^2^: %HRmax (average HR/Predicted HR max) during 20-km run.

^3^: Energy expenditure during endurance exercise = Maximal energy expenditure (kcal/min)* [(% HRmax) * 1.4301–47.755]*time (min)

### Phenylalanine flux

**Fig 2:**
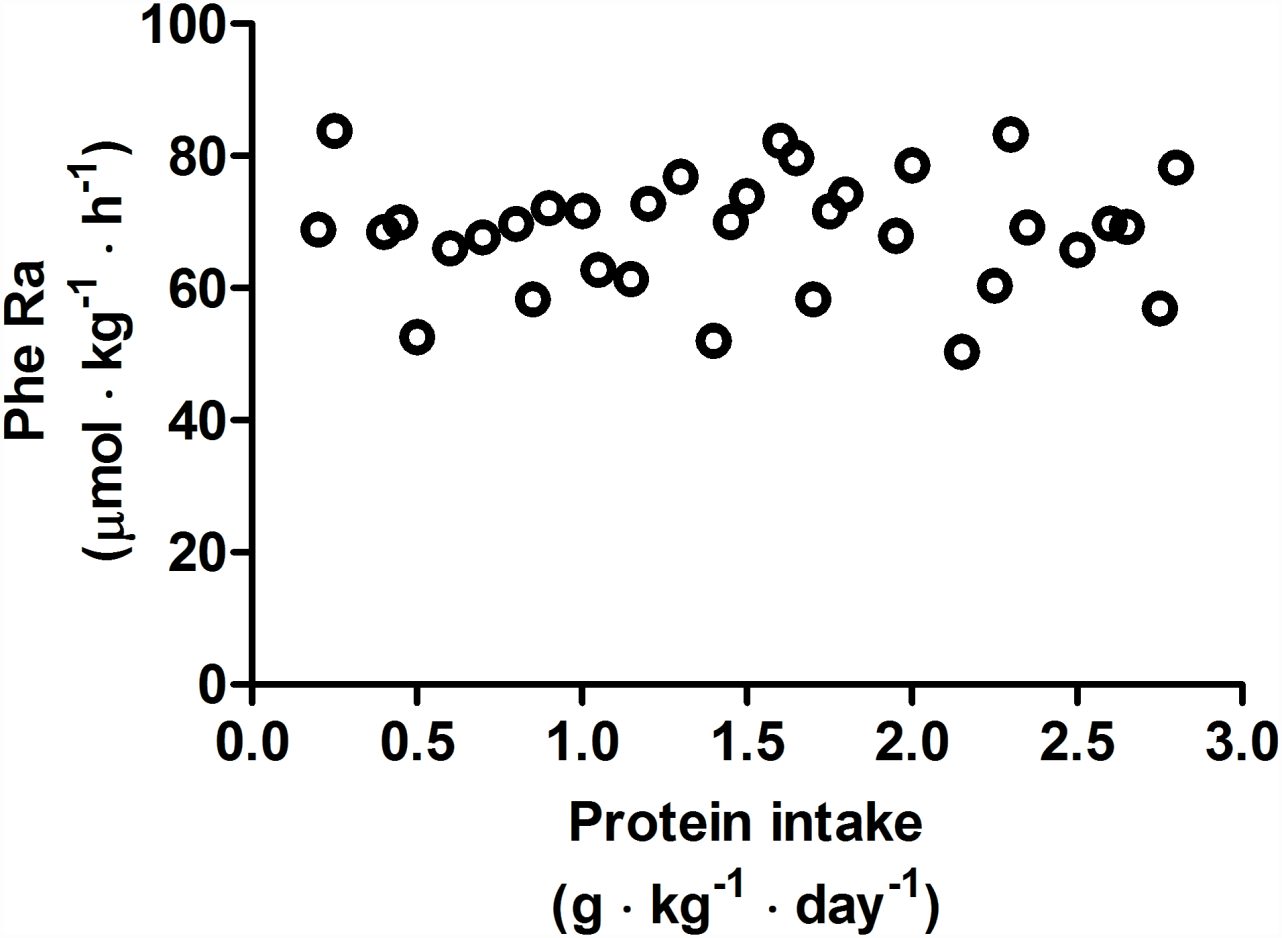
Relationship between Phenylalanine Ra and protein intake after exercise stimulus. Each data point represents PheRa on the individual metabolic trial day. The slope of regression line was not significantly different from zero (P = 0.11).

Phenylalanine flux was not affected by protein intake (Fig 2, P = 0.11, average phenylalanine flux = 68.8±8.5 μmol·kg^-1^·h^-1^) and was stable within each participant (Table 5). This indicated that the phenylalanine pool for the IAAO did not change in response to increasing test protein intakes, which would suggest that the change in phenylalanine oxidation reflect whole-body protein synthesis [13].

**Table 5:** The effect of protein intake on phenylalanine fluxes. Values are means ± SD. No significant differences (P > 0.05) in phenylalanine flux were observed within each participant because of various test protein intakes.

Subject No.	Phenylalanine flux (μmol·kg^-1^·h^-1^)
1	65.3 ± 5.7
2	74.1 ± 6.3
3	63.5 ± 10.7
4	73.7 ± 7.2
5	74.8 ± 7.2
6	64.6 ± 7.6

### Average protein requirement and recommended protein intake

**Fig 3:**
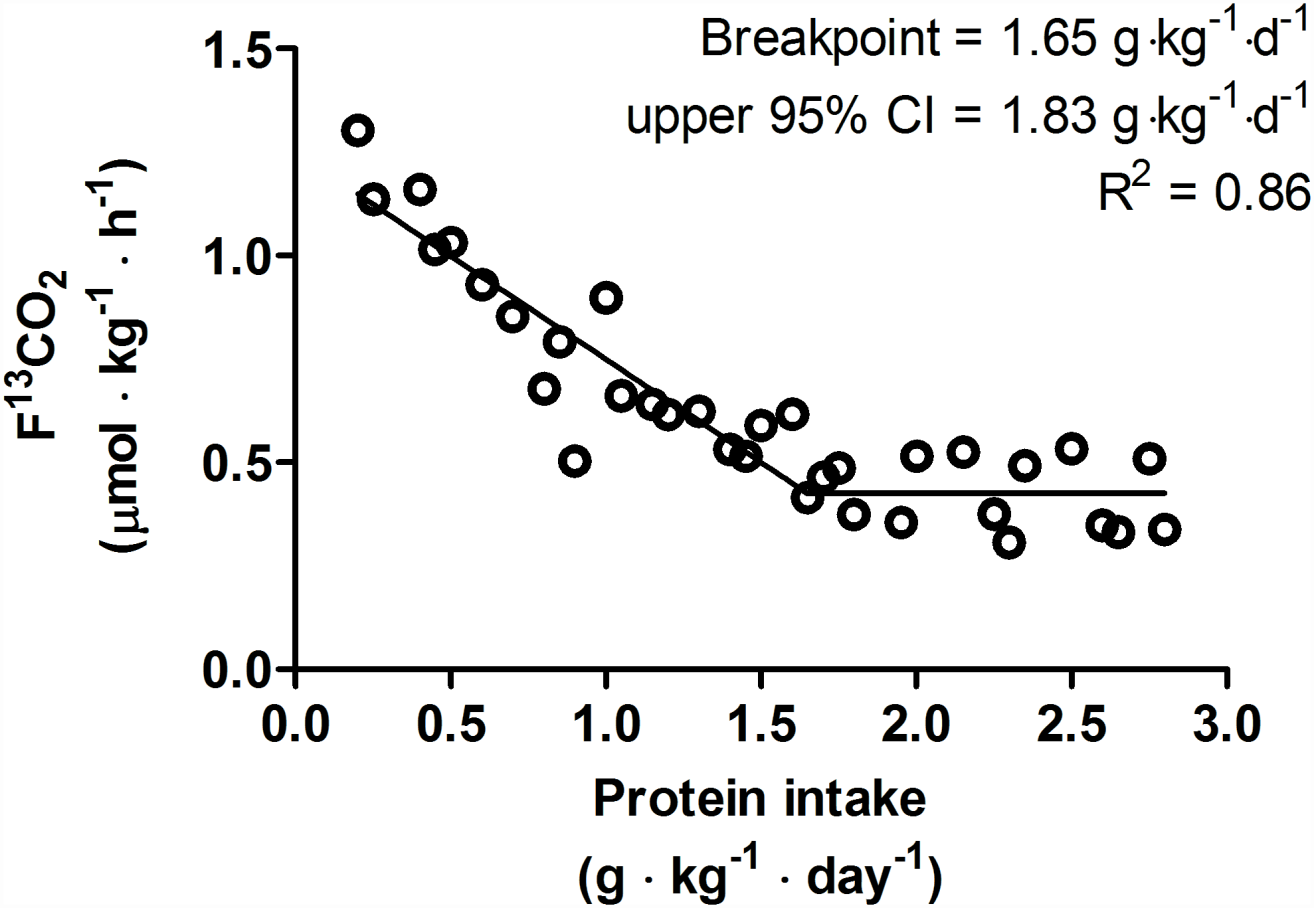
Relationship between protein intake and F^13^CO_2_. 6 participants completed 34 metabolic trials with a range of test protein intake (0.2–2.8 g·kg^-1^·d^-1^). The breakpoint represented the average protein requirement. The breakpoint was determined by using a biphasic linear regression crossover analysis. The average protein requirement and recommended protein intakes were estimated to be 1.65, 1.83 g·kg^-1^·d^-1^ respectively (R^2^ = 0.86).

**Fig 4:**
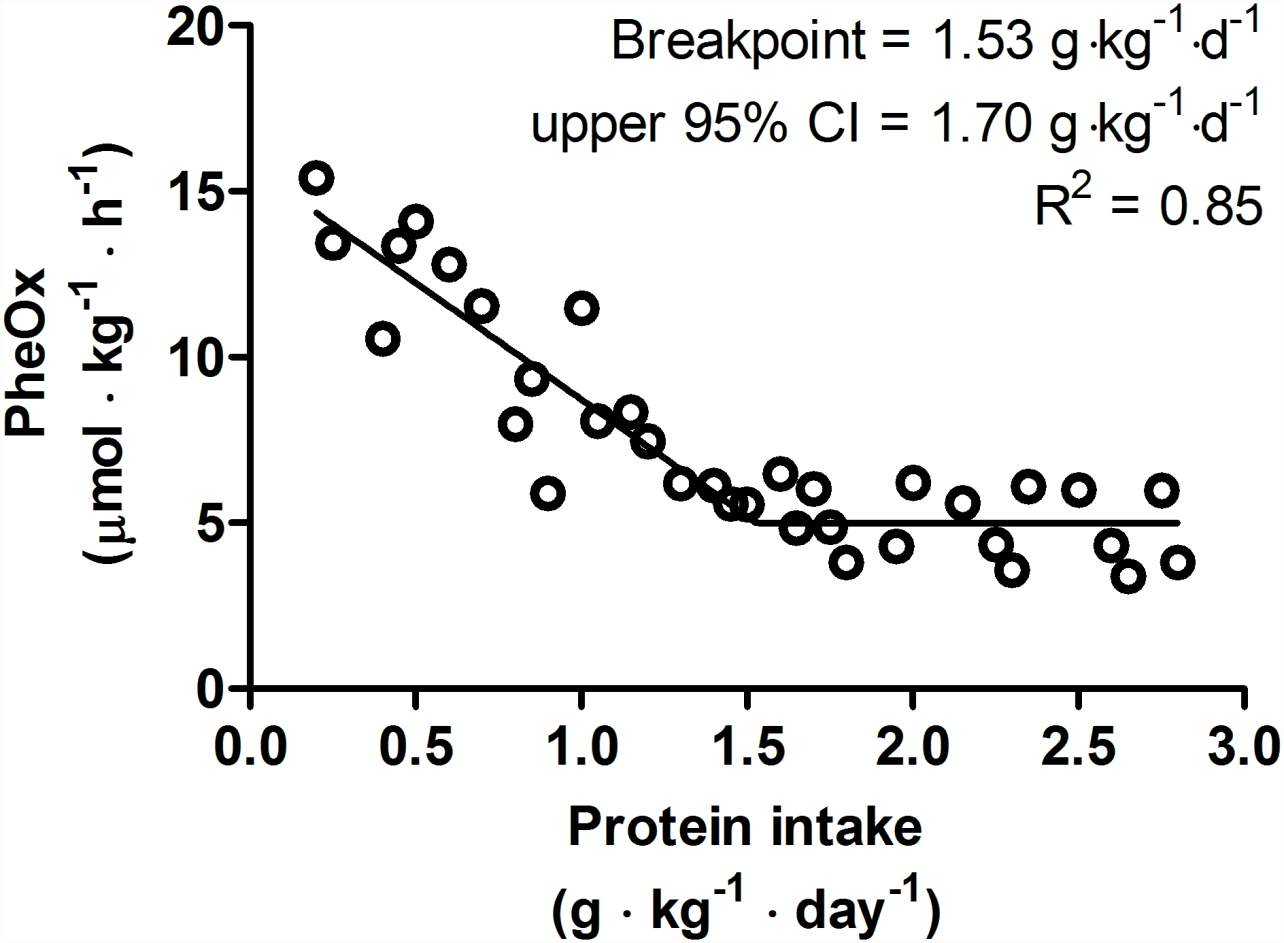
Relationship between protein intake and PheOx. 6 participants completed 34 metabolic trials with a range of test protein intake (0.2–2.8 g·kg^-1^·d^-1^). The breakpoint estimated the average protein requirement. The breakpoint was determined by using a biphasic linear regression crossover analysis. The average protein requirement and recommended protein intake were estimated to be 1.53, 1.70 g·kg^-1^·d^-1^ respectively (R^2^ = 0.85).

Biphasic linear regression crossover analysis (R^2^ = 0.86) of F^13^CO_2_ revealed a breakpoint (i.e. average protein requirement) at 1.65 g·kg^-1^·d^-1^ (Fig 3). The upper 95% CI (i.e. recommended protein intake) was determined to be 1.83 g·kg^-1^·d^-1^. Subsequently, a biphasic linear regression crossover analysis (R^2^ = 0.85) of PheOx revealed a breakpoint at 1.53 g·kg^-1^·d^-1^ (Fig 4). The upper 95% CI was determined to be 1.70 g·kg^-1^·d^-1^. Although the average protein requirements and recommended protein intakes were similar between the breakpoint analyses of F^13^CO_2_ and PheOx, F^13^CO_2_ data are generally considered more robust as they more closely align with rates of phenylalanine hydroxylation determined from apolipoprotein B-100 enrichment and, hence, is reflective of the true intracellular enrichment for protein synthesis [28, 31].

## Discussion

The objective of this study was to investigate the estimated average protein requirement and recommended protein intake in endurance-trained individuals utilizing, for the first time in an active population, the minimally-invasive IAAO method. Our data revealed that within a simulated controlled training program (i.e. 35 km over 3 days) and on a day in which an acute bout of endurance exercise (i.e. 20-km run) is performed, the estimated average protein requirement and recommended protein intake (as determined by upper 95% CI) were 1.65 and 1.83 g·kg^-1^·d^-1^, respectively, in our endurance trained population; these values, which represent a modest ~12% of total energy intake in the present study, are greater than the current RDA [1] determined by NBAL technique [32] and IAAO method [7] in non-exercised individuals.

We provided carbohydrate intakes (~9 g/kg/d) in accordance with general sport-specific recommendations to replenish glycogen stores in endurance athletes [20]. Although carbohydrates (and the associated insulin response) may reduce whole body protein breakdown after exercise [33] and enhance nitrogen retention at rest [34], phenylalanine flux in the present study (~69 μmol·kg^-1^·d^-1^/kg/d) was ~17% higher than that previously observed in non-exercised young adults (~59 μmol·kg^-1^·d^-1^/kg/d) at rest [7] using identical methodology; this difference is attenuated slightly when data from both studies are normalized to the metabolically active lean tissue mass (i.e. ~79 μmol·kg FFM^-1^·d^-1^ vs. ~72 μmol/·kg lean body mass(LBM)^-1^ ·d^-1^, respectively). While we cannot identify the potential site(s) of altered protein turnover in our whole body model, previous studies have shown that muscle protein turnover is increased in aerobically-trained individuals [35] and that acute exercise elevates protein breakdown [36] after endurance exercise due to an activation of the protein degradation systems [37]. Whether these factors may have influenced the slightly higher phenylalanine flux in the present study relative to that previously reported in non-exercising adults is unclear [7]. Nevertheless, the lack of effect of protein intake on phenylalanine flux permits the reliable estimation of the recommended protein intake from the F^13^CO_2_ breakpoint [7, 12, 13, 25].

According to sports nutrition consensus statements mainly based data from NBAL studies, protein recommendations for endurance athletes have been suggested to be 1.2–1.4 g protein/kg/d [1]; these recommendations are 50–75% greater than the current RDA of 0.8 g/kg/d. In the present study, the recommended protein intake was determined to be 1.83 g·kg^-1^·d^-1^, which is ~31–53% greater than previous recommendations for endurance trained populations on the basis of NBAL data [1]. The differences in protein recommendations may be related to the methodology employed (i.e. NBAL vs. IAAO) [38, 39] and therefore our data is perhaps more accurately compared to previous studies utilizing the same methodology. In this case, our average protein requirement and recommended protein intake are ~77 and ~53%, respectively, greater than those previously determined by IAAO method in non-exercised adults [7]. Therefore, our data are generally consistent with previous literature suggesting that endurance exercise increases protein requirements but suggest that intakes ~120% greater than the current RDA and at the upper end of general protein recommendations for athletes (i.e. 1.2–2 g·kg^-1^·d^-1^) [40] may be required to maintain protein balance.

The average protein requirement during a period of controlled training (i.e. 35 km over 3 days) and after a 20-km run was determined to be 1.65 g·kg^-1^·d^-1^, which is ~77% higher than the average protein requirement (0.93 g·kg^-1^·d^-1^) in healthy adults determined by IAAO methodology [7] and represents a relative difference between physiological states of ~0.72 g·kg^-1^·d^-1^. In the present study, participants expended ~1100 kcal during exercise. Assuming that amino acid oxidation contributes ~5% of total energy expenditure during exercise [41], this exercise-induced amino acid oxidation could have resulted in ~14 g or the equivalent of ~0.2 g·kg^-1^·d^-1^ of total protein be irreversibly oxidized. Furthermore, if exercise is performed under conditions of low muscle glycogen, the contribution of protein to total exercise energy expenditure may be as high as ~10% [41]. Given that our participants were exercising at an intensity (~74%HRmax) that would have relied heavily on muscle glycogen as a source of fuel [42, 43] and for a duration (~99 min) that would have resulted in significant depletion of endogenous stores [44], it is possible that the exercise-induced protein catabolism was greater than ~0.2 g·kg^-1^·d^-1^. Therefore, oxidative protein losses may have explained at least ~31% (but likely more) of the greater average protein intake in the present study relative to non-exercised adults [7] and, similar to previous suggestions [4], could have contributed to the greater protein requirements in our endurance athlete population.

In addition to the oxidative losses of body amino acids, endurance exercise is also a major stimulus to remodel and repair a variety of body proteins. For example, endurance exercise can enhance the degradation of skeletal muscle proteins during exercise [45] and stimulate muscle protein synthesis for up to 24h after exercise [37, 46], the latter of which is enhanced with dietary protein ingestion [47]. Inasmuch as this enhanced muscle tissue remodeling may function to repair acute muscle damage, the running modality in the present study may have provided a greater stimulus for muscle remodeling than previous studies employing a cycling modality [48]. In addition to enhancing the synthesis of muscle and plasma (i.e. albumin) protein synthesis during recovery [49], endurance exercise (i.e. 1h of cycling) has been reported to increase markers of intestinal damage through a potential ischemia-reperfusion mechanism [48]. Whether this exercise-induced gut damage would be greater in weight-bearing exercise (e.g. due to acceleration/deceleration forces associated with running), result in an increased splanchnic protein turnover, and/or require exogenous dietary amino acids to aid in the repair is currently unknown. Therefore, endurance exercise (perhaps especially that which is weight-bearing) may induce remodeling and/or repair of a variety of body proteins that presently have unknown consequences on protein requirements but may have contributed to the greater average protein requirement and recommended protein intake in our trained athletes relative to non-exercised individuals [7].

We studied participants after a 20 km training session (but within a 3-d controlled training period) as we believe that the exercise-induced increase in amino acid oxidation and the acute stimulation of post-exercise protein remodeling would be the factors that would most likely increase protein requirements in this athlete population. The exercise load (20-km run) and intensity was selected to provide a stimulus that would presumably induce elevated peroxisome proliferator-activated receptor-gamma coactivator 1α expression/activity [50] and enhance mitochondrial protein synthesis [50, 51] as well as be reflective of the habitual training of a variety distance runners aiming to augment aerobic adaptations. Moreover, given the frequency with which endurance athletes generally train it is likely that most (if not all) days of the week would incorporate some sort of exercise training [52, 53], which further influenced our decision to study athletes on a day in which they performed exercise. Inasmuch as the greater requirements in the present study were the result of an increased oxidative disposal of amino acids during exercise, our results could suggest that training days with greater exercise volume (i.e. those requiring greater total oxygen consumption [54]) may require slightly greater protein requirements with the reverse being true for lower volume training days. The potentially greater contribution of endogenous protein to energy provision during periods of low glycogen availability [41, 45] could suggest that contemporary periodized training approaches featuring periods of low carbohydrate availability training to enhance metabolic (e.g. fat oxidation) and/or aerobic (i.e. mitochondrial biogenesis) adaptations [55] may also require greater protein intakes.

Interestingly, females have been reported to have a lower reliance on amino acid oxidation as a fuel source due to the protective effects of estrogen [4, 56]. The protein-sparing effect of estrogen could suggest their protein requirements within a controlled training period and after a similarly intense 20-km run could be lower than those determined in the present study in males, as has been suggested previously in trained cyclists [57, 58]. Ultimately, additional work is required to elucidate whether (and to what extent) different training volumes, intensities, modalities (e.g. cycling vs running), and/or nutritional manipulations (e.g. high vs. low carbohydrate availability, low energy availability) may influence protein requirements in different athletic populations (e.g. males and females).

## Conclusion

In conclusion, we report using the novel IAAO method that endurance-trained athletes consuming adequate energy and carbohydrate during a controlled training period have a greater recommended protein intake than those previously established in endurance-trained adults by NBAL and sedentary adults by IAAO. Our estimates of the average (1.65 g/kg/d) and recommended intakes (1.83 g/kg/d) for protein are generally within the habitual intake of male (but perhaps not female) endurance trained populations [4, 59]; however, it is unclear if these daily protein targets are “optimal” with respect to health and/or performance outcomes for these athletes. Therefore, our results could provide the framework from which future studies could elucidate whether protein intakes that deviate substantially from those determined herein confer any ergogenic benefits or have any ergolytic consequences.

## Supporting Information

S1 CONSORT Checklist CONSORT checklist. (DOCX)

S1 Protocol Study protocol containing background, hypothesis, outcome parameters and experimental design. (PDF)

S1 Text The pilot study to determine an isotopic and metabolic steady state during metabolic trial. (DOCX)
