# Supplementary material for: Protein Requirements Are Elevated in Endurance Athletes after Exercise as Determined by the Indicator Amino Acid Oxidation Method
Source: PLoS One. 2016 Jun 20;11(6):e0157406. doi: 10.1371/journal.pone.0157406 (PMC4913918; doi:10.1371/journal.pone.0157406)
Supplement: S1 Protocol — (PDF) [file pone.0157406.s002.pdf]

UNIVERSITY OF TORONTO

Office Use Only
Protocol Number:

Office of the Vice-President, Research and Innovation

Office of Research Ethics

ETHICS REVIEW PROTOCOL SUBMISSION FORM FOR SUPERVISED AND SPONSORED RESEARCHERS

(For use by graduate students, post-docs, and visiting professors/researchers)

SECTION A – GENERAL INFORMATION

1. TITLE OF RESEARCH PROJECT

Evaluation of protein requirements in male endurance athletes

2. INVESTIGATOR INFORMATION

Investigator:

Title (e.g., Dr., Ms., etc.): Mr.	Name: Hiroyuki KATO
Department (or organization if not affiliated with U of T): Faculty of Kinesiology and Physical Education	
Mailing address: 55 Harbord St., Toronto, ON M5S 2W6	
Phone: 416-946-5620	Institutional e-mail: h.kato@utoronto.ca

Level of Project:

Student Research: Doctoral ☐	Masters ☐	
Post-Doctoral Research ☐	Visiting professor/researcher ☒	Course Based ☐
CBR/CBPR ☐	Other ☐ (specify:)	

Faculty Supervisor/Sponsor:

Title: Dr.	Name: Daniel Moore
Department (or organization if not affiliated with U of T): Faculty of Kinesiology and Physical Education	
Mailing address: 55 Harbord St., Toronto, ON M5S 2W6	
Phone: 416-946-4088	Institutional e-mail: dr.moore@utoronto.ca

Co-Investigators:

Are co-investigators involved? Yes ☐ No ☒

Title:	Name:
Department (or organization if not affiliated with U of T):	
Mailing address:	
Phone:	Institutional e-mail:

Title:	Name:
Department (or organization if not affiliated with U of T):	
Mailing address:	
Phone:	Institutional e-mail:

Title:	Name:
Department (or organization if not affiliated with U of T):	
Mailing address:	
Phone:	Institutional e-mail:

Please append additional pages with co-investigators' names if necessary.

3. UNIVERSITY OF TORONTO RESEARCH ETHICS BOARD:

Health Sciences ☒ Social Sciences, Humanities and Education ☐ HIV/AIDS ☐

To determine which Research Ethics Board (REB) your protocol should be submitted, please consult: http://www.research.utoronto.ca/for-researchers-administrators/ethics/human/boards-committees/..

4. LOCATION(S) WHERE THE RESEARCH WILL BE CONDUCTED:

If the research is to be conducted at a site requiring administrative approval/consent (e.g., in a school), please include all administrative consent letters. It is the responsibility of the researcher to determine what other means of approval are required, and to obtain approval prior to starting the project.

University of Toronto ☒
Hospital ☐ specify site(s)
School board or community agency ☐ specify site(s)
Community within the GTA ☐ specify site(s)
International ☐ specify site(s)
Other ☐ specify site(s)

The University of Toronto has an agreement with the Toronto Academic Health Sciences Network (TAHSN) hospitals regarding ethics review of hospital-based research where the University plays a peripheral role. Based on this agreement, certain hospital-based research may not require ethics review at the University of Toronto. If your research is based at a TAHSN hospital please consult the following document to determine whether or not your research requires review at the University of Toronto. http://www.research.utoronto.ca/for-researchers-administrators/ethics/human/at-a-glance/where-to-apply-tahsn-institutions/

5. OTHER RESEARCH ETHICS BOARD APPROVAL(S)

(a) Does the research involve another institution or site? Yes ☒ No ☐
(b) Has any other REB approved this project? Yes ☒ No ☐
If **Yes**, please provide a copy of the approval letter upon submission of this application.
If **No**, will any other REB be asked for approval?
Yes ☐ (please specify which REB) No ☐
Please note that REB approvals from other sites must be submitted to the ORE at U of T

6. FUNDING OF THIS PROJECT

(a)

Funding Status	Source and Type	Details
Funded ☒	Sponsored Research and Collaboration	Fund #:
	Agency:	Fund # : (6 digits)
Applied for funding ☐	Agency:	Submission date:
	Agency:	Submission date:
Unfunded ☐<br>If unfunded, please explain why no funding is needed:		

(b) If one protocol is to cover more than one grant, please include all fund numbers:

7. CONTRACTS

Is this research to be carried out as a contract? Yes ☒ No ☐

If yes, is there a University of Toronto funding or non-funded agreement associated with the research?
Yes ☒ No ☐
If **Yes,** please append a copy of the agreement with of this application.

Is there any aspect of the contract that could put any member of the research team in a potential conflict of interest? Yes ☐ No ☐
If yes, please elaborate under #10.

8. PROJECT START AND END DATES

Estimated start date for the component of this project that involves human participants or data: Febrary 1, 2015
Estimated completion date of involvement of human participants or data for this project: Febrary 1, 2016

9. SCHOLARLY REVIEW:

(Please note: for submissions to the **HIV REB** from community investigators, scientific review is a pre-requisite for ethics review. If your study is unfunded, please contact the OHTN to arrange a scientific review prior to completing your ethics submission.)

(a) Please check one:

I. ☒ The research has undergone scholarly review by thesis committee, departmental review committee, peer review committee or some other equivalent (Specify review type – e.g., departmental research committee, supervisor, CIHR, SSHRC, OHTN, etc.): Departmental Committee Review
II. ☐ The research will undergo scholarly review prior to funding (Specify review committee – e.g., departmental research committee, SSHRC, CIHR peer-review committee, etc.):
III. ☐ The research will not undergo scholarly review (Please note that all research greater than minimal risk requires scholarly review)

(b) If box I or II above was checked, please specify if:

☒ The review was/will be specific to this protocol

☐ The review was/will be part of a larger grant

10. CONFLICTS OF INTEREST

(a) Will the researcher(s), members of the research team, and/or their partners or immediate family members:
(i) Receive any personal benefits (e.g., financial benefit such as remuneration, intellectual property rights, rights of employment, consultancies, board membership, share ownership, stock options, etc.) as a result of or in connection with this study? Yes ☐ No ☒
(ii) If **Yes**, please describe the benefits below. (Do not include conference and travel expense coverage, or other benefits which are considered standard for the conduct of research.)

No Benefits

(b) Describe any restrictions regarding access to or disclosure of information (during or at the end of the study) that have been placed on the investigator(s). These restrictions include controls placed by the sponsor, funding body, advisory or steering committee.

The University will provide a copy of any proposed publication of Project research results (a "Publication") to the Sponsor for its review at least sixty (60) days before submission for publication or disclosure. Upon the Sponsor's written request received within sixty (60) days of the Sponsor's receipt of the Publication, the University will, at the Sponsor's option:

a. delete identifiable references to any Confidential Information provided by the Sponsor from the proposed Publication;

b. if the Sponsor has exercised the Option, delay publication of the Publication up to thirty (30) additional days to enable the Sponsor to file, in the name of the IP owner or its assignee(s), patent application(s) for any Intellectual Property that would be publicly disclosed in the Publication.

The Sponsor cannot withhold findings nor request further study.

(c) Where relevant, please explain any pre-existing relationship between the researcher(s) and the researched (e.g., instructor-student; manager-employee; clinician-patient; minister-congregant). Please pay special attention to relationships in which there may be a power differential – actual or perceived.

No Pre-Existing Relationships

(d) Please describe the decision-making processes for collaborative research studies. If Terms of Reference exist, attach them. Collaborative research studies include those where a number of sites (e.g. other universities, non-TAHSN hospitals, etc.) are involved, as well as those that involve community agencies.

All of the participant testing and analysis will be conducted at Goldring Centre for High Performance Sport and at the Athletic Centre within University of Toronto Upon completion of breath and urine analysis, the results of the breath and urine samples will be provided to the research team at the University of Toronto for further analysis. Confidentiality of the participants will be maintained as all participants will be provided with a subject number, and all data will be stored securely. Samples will be coded at the University of Toronto prior to being analyzed. Subject codes will also only be stored at the University of Toronto. The research team at the University of Toronto will make any decisions pertaining to the exercise protocol. Any decisions pertaining to the implementation of the IAAO technique, or study diets will be made in the metabolic kitchen in the Goldring Centre for High Performance Sports. The IAAO study diet protocol will be adhered to in its entirety.

SECTION B – SUMMARY OF THE PROPOSED RESEARCH

11. RATIONALE

Describe the purpose and scholarly rationale for the proposed project. State the hypotheses/research questions to be examined. The rationale for doing the study must be clear. Please include references in this section.

Provided energy needs are met, the adequate ingestion of dietary amino acids is the most critical nutritional factor to support the optimal remodeling and deposition of lean body mass in individuals of all ages. Of primary importance is the impact exercise has on the nutritional requirement for dietary amino acids in highly active adults. Current recommendations according to the World Health Organization/Food and Agricultural Organization suggest that daily protein requirements in healthy, non-active adults are 0.8 g/kg/day. Protein requirements in individuals who participate in endurance-based exercise training have been suggested to be 40-100% greater than the current RDA (Tarnopolsky, 2004), which may reflect the requirement for protein to

repair/rebuild lean tissues and to replace oxidative losses (Moore, Camera, Areta, and Hawley, 2014). Nutritional requirements for dietary amino acids in adults (both active and non-active) have traditionally been determined utilizing the antiquated and often erroneous nitrogen balance technique, which is prone to overestimating protein requirements and therefore provides challenges to making accurate nutritional recommendations (Humayun, Elango, Ball, and Pencharz, 2007). As a result, there is a need to re-evaluate recommendations utilizing advanced stable isotope methodology in order to characterize how dietary amino acid needs may be modulated by physical activity. Recent studies using the minimally invasive indicator amino acid oxidation (IAAO) technique have suggested that protein requirements in young men are at least 50% higher than WHO/FAO guidelines based on nitrogen balance data (Humayun, Elango, Ball, and Pencharz, 2007; Elango, Humayun, Ball, and Pencharz, 2007). Given that endurance training has been reported to increase (according to nitrogen balance methodology) protein requirements by up to 100% over untrained individuals (Tarnopolsky, 2004), this observation that nitrogen balance underestimates protein requirements in non-active individuals could suggest that protein requirements are much greater than the current WHO/FAO recommendation of 0.8 g/kg/day. Therefore, in this study, we will use the gold standard IAAO technique to determine protein requirements in endurance-trained adults. It is hypothesized that the present study will deem current reveal protein requirements are greater than the current RDA for non-active individuals comparable estimates (as determined by nitrogen balance) in active, endurance trained adults.

REFERENCES

Boisseau N, Le CC, Loyens M, and Poortmans JR. Protein intake and nitrogen balance in male non-active adolescents and soccer players. Eur J Appl Physiol. 88: 288-293, 2002.

Boisseau N, Persaud C, Jackson AA, and Poortmans JR. Training does not affect protein turnover in pre- and early pubertal female gymnasts. Eur J Appl Physiol. 95: 262-267, 2005.

Boisseau N, Vermorel M, Rance M, Duche P, and Patureau-Mirand, P. Protein requirements in male adolescent soccer players. Eur J Apply Physiol. 100: 27-33, 2007.

Elango R, Ball RO, and Pencharz PB. Recent advances in determining protein and amino acid requirements in humans. Br J Nutr. 198 Suppl 2: S22-S30, 2012.

Elango R, Humayun MA, Ball RO, and Pencharz PB. Protein requirement of healthy school-age children determined by the indicator amino acid oxidation method. Am J Clin Nutr 94: 1545-1552, 2011.

Humayun MA, Elango R, Ball RO, and Pencharz PB. Reevaluation of the protein requirement in young men with the indicator amino acid oxidation technique. Am J Clin Nutr. 86: 995-1002, 2007.

Moore DR, Camera DM, Areta JL, and Hawley JA. Beyond muscle hypertrophy: why dietary protein is important for endurance athletes. Applied physiology, nutrition, and metabolism = Physiologie appliquee, nutrition et metabolisme 39: 987-997, 2014

Tarnopolsky M. Protein requirements for endurance athletes. Nutrition 20: 662-668, 2004.

12. METHODS

(a) Please describe all formal and informal procedures to be used. Describe the data to be collected, where and how they will be obtained and how they will be analyzed.

<u>Evaluation of protein requirements in endurance athletes (refer to protocol in appendix):</u>

Participants will be recruited by way of flyer or word-of-mouth (see Section 15 for recruitment details).
This study contains “main study” and “pilot study”.
In main study,utilization of the IAAO technique has been used extensively to estimate dietary indispensable amino acid and protein requirement with 5-8 young adult males and females consuming 7 different test protein intakes (i.e. a total of ~35-56 metabolic trials). A target of 8 young adult males will be recruited for the main study. Total participant time commitment will span over 9 sessional dates and have an approximate total time commitment of 74.5 hours (7 x 10h, + 1 x 1.5h, + 1 x 4h). Prior to all sessions, participants will have

abstained from alcohol consumption for 48 hours, will be encouraged to have slept at least 7 hours the night before, and will not have consumed any caffeine that day. For 2-days before each metabolic trial, participants will consume the maintenance-diet (see additional information in appendix.), participants will be required to perform standard exercise on 2 days before the metabolic trials, which will involve a 10-km run on the first day and a 5-km run on the second day (according to a self-selected running pace).

Main study

Session One - Introduction (~1.5h)

Recruited participants will come into the Iovate/Muscletech Metabolism and Sports Science Lab (I/M Lab) at the Goldring Centre for High Performance Sport to first engage in an introductory session. Participants will not be admitted to the study until after this session has finished, and no data collection will be recorded or saved unless participants have been committed to the study. Participants will be given a comprehensive oral introduction to the study by a co-investigator to explain the study objectives, conduct, and associated risks. Prior to asking for written consent, the study will be explained in detail to the participants. Demonstrations of the use of accelerometers, BodPod, HR-monitors, breath and urine sampling, and the study diet protocol will be provided to the participants prior to asking for their commitment. Participants will not be asked to fill out any surveys, or to partake in any testing prior to signing consent forms. Participants will be given an opportunity to read consent forms, and to ask questions pertaining to the study (see section 19). Session one will then serve to collect anthropometric measures, and to screen for habitual activity levels using the PAR-Q+ and the survey sheet for training log (see appendix). Participants that meet the inclusion criteria and doesn't meet the exclusion criteria will be enrolled in the study. Participants will also be fitted with the Sensewear Body Media Armband Accelerometer to be worn for 3 days prior to Session Two. This will provide the co-investigators with an estimate of all participants' free-living energy expenditure. Participants will be instructed on how to complete a 3-d food record by co-investigator to determine habitual dietary intake prior to Session Two (see appendix).

Session Two – Body Composition Measurements, Fitness Assessment (~4h)

Participants that have been enrolled to the study will report to the I/M Lab having worn the Sensewear Body Media Armband Accelerometer for 3 days, having completed a 3-d dietary record after overnight fasting. BodPod analysis will be conducted to determine the baseline whole body, fat mass (FM) and fat-free mass (FFM) of each participant; this will be done to normalize data to both whole body mass and lean body mass. Resting energy expenditure will be measured by gas exchange using Moxus Metabolic Cart (AEI Technologies). Participants will then be familiarized with the fitness assessment protocol to ensure they are comfortable with all equipment. A treadmill-based ramp protocol exercise test will be used to determine the participants' maximal oxygen consumption (VO_2-max). Participants will be instructed to continue to wear the Sensewear Body Media Armband Accelerometer throughout the duration of the fitness assessment. Subjects will be equipped with a ECG electrodes to measure heart rate, a blood pressure cuff, and a mask to measure all inhaled and exhaled air. Gas exchange data will be will be analyzed using a Moxus Metabolic Cart (AEI Technologies). The treadmill-based fitness assessment will consist of a 12-minute test, which has previously been applied and established in young adults (see appendix) and will cover an estimated distance of ~4km. Subjects will then be given a 30-minute rest prior to engaging in the exercise familiarization trial.

Upon completion of the treadmill-based fitness assessment, all participants will be provided with a brief explanation of the testing protocol prior to performing a 16-km run on a treadmill in the Goldring centre for High Performance Sport. This will ensure that the participants are both familiar with the testing protocol on metabolic trial sessions and that they are able to complete 20-km run successfully. Additionally, this will provide us with an estimate of their exercise-induced energy expenditure to ensure the diet will provide sufficient energy on the trial day.

Sessions 3 to 9 – Metabolic Trials (~10h each; at least 4d washout between trials)

For 2-days before each of the seven metabolic trials, all young adult participants will be required to consume a diet providing 1.5g of protein/kg/d with energy to cover the resting energy expenditure (REE) and exercise-induced energy expenditure, the latter of which will be monitored during the 3-d assessment period by the accelerometer. This level of dietary protein is selected to meet the protein requirements of adults. The young adults will consume a controlled diet provided by the investigators that will be provided in the form of the commercially available, pre-packaged foods, which are similar to what the participants normally eat. All vitamin and mineral requirements will be met with a daily multivitamin.

Bod pod Analysis will be measured on the 4th and 7th trial days or every month (whichever comes first) to monitor potential changes in lean mass throughout the study protocol. Upon waking, participants will consume a protein-free liquid drink providing 300 kcal of energy (75 g carbohydrate) 1h before reporting to the Goldring Centre for High Performance Sport within University of Toronto. Upon arrival, participants will perform a brief 10 min warm up run before completing a 20 km run at a self-selected race pace. The study diet will consist of 8 hourly meals beginning immediately after the 20-km run. The amino acid pattern of the test protein intake will be modeled on the basis of egg protein, with the exception of phenylalanine (the indicator amino acid) and tyrosine. Phenylalanine will be held constant at an intake of 30.5 mg/kg/d and tyrosine will be held constant at 40 mg/kg/d. Tyrosine will be provided in excess in order to accurately measure the oxidation of the indicator amino acid. All participants will be randomly assigned to ingest 7 different test protein intakes in a random order on each of the seven metabolic trial days that will have a range designed to cover deficient and excessive intake (i.e. 0.1-2.8 g/kg/d) as suggested by previous protein requirements determined by IAAO in young adults. Each of these trials will be separated by a minimum of 4 days washout. A minimum number of four metabolic trials will be required from all enrolled participants to ensure that a total of metabolic trials within the range of ~35 and 56 is satisfied.

Directly after the 20-km run on each metabolic trial day, the participants will consume the first of 4 hourly meals according to the test protein intake indicated above. Prior to the initiation of the oral tracer ingestion, 3 breath samples will be taken at 15 minute intervals, and 2 urine samples will be collected at 30 minute intervals to determine baseline $^{13}CO_2$ and L-[^{13}C]phenylalanine enrichment respectively. On the 5th meal, a priming dose of $NaH^{13}CO_3$ (0.176 mg/kg) and L-[^{13}C]phenylalanine (0.66 mg/kg) will be ingested. The rate of CO_2 production will then be measured over a 20-minute period immediately after the 5th meal via indirect calorimetry. Subsequently, 1.22 mg/kg of L-[^{13}C]phenylalanine will be ingested in each hourly meal to maintain isotopic steady state until the end of the metabolic trial. Four plateau breath and urine samples will be collected at 30min intervals beginning 2.5h after the onset of tracer ingestion. Breath $^{13}CO_2$ enrichment will be measured by continuous-flow isotope ratio mass spectrometry and urinary L-[^{13}C]phenylalanine by gas chromatography-mass spectrometry.

A mixed linear model will be used to determine the effect of protein intake on $F^{13}CO_2$, phenylalanine flux, and phenylalanine oxidation in line with previous studies (see appendix). Significant differences between means will be assessed using a Tukey-Kramer's multiple comparisons test, where appropriate. Significance will be set at $P < 0.05$. To determine the mean protein requirement, a bi-phase linear regression cross-over analysis will be performed on $F^{13}CO_2$ (as the primary outcome) and phenylalanine oxidation (as a secondary outcome) in agreement with previous studies. A safe protein intake will be estimated by the upper 95% CI of the mean requirement breakpoint.

Prior to main study, the pilot study will be done. The objective of the pilot study is to ensure that CO2 production and 13O2 enrichment reaches a plateau after endurance run. A target of 4 young adult males will be recruited for the pilot study. Total participant time commitment will span over 3 sessional dates and have an approximate total time commitment of 13.5 hours (7 x 8 h, + 1 x 1.5h, + 1 x 4h). All condition except for session 3 are same as the main study, in pilot study.

Pilot study

Session 3 – Metabolic Trials (~10h)

For 2-days before each of the seven metabolic trials, all young adult participants will be required to consume a diet providing 1.5g of protein/kg/d with energy to cover the resting energy expenditure (REE) and exercise-induced energy expenditure, the latter of which will be monitored during the 3-d assessment period by the accelerometer. This level of dietary protein is selected to meet the protein requirements of adults. The young adults will consume a controlled diet provided by the investigators that will be provided in the form of the commercially available, pre-packaged foods, which are similar to what the participants normally eat. All vitamin and mineral requirements will be met with a daily multivitamin.

Upon waking, participants will consume a protein-free liquid drink providing 300 kcal of energy (75 g carbohydrate) 1h before reporting to the Goldring Centre for High Performance Sport within University of Toronto. Upon arrival, participants will perform a brief 10 min warm up run before completing a 20 km run at a self-selected race pace. The study diet will consist of 8 hourly meals beginning immediately after the 20-km run. The amino acid pattern of the test protein intake will be modeled on the basis of egg protein, with the exception of phenylalanine (the indicator amino acid) and tyrosine. Phenylalanine will be held constant at an

intake of 30.5 mg/kg/d and tyrosine will be held constant at 40 mg/kg/d. Tyrosine will be provided in excess in order to accurately measure the oxidation of the indicator amino acid.
Directly after the 20-km run, the participants will consume 8 hourly meals, 18 breath samples will be taken at 30 minutes intervals and VCO2 will be measured for 20 minutes at 1hour intervals to determine baseline 13CO2 enrichment and VCO2 respectively.
Breath 13CO2 enrichment will be measured by continuous-flow isotope ratio mass spectrometry and urinary L-[13C]phenylalanine by gas chromatography-mass spectrometry.

(b) Attach a copy of all questionnaires, interview guides and/or any other instruments.

(c) Include a **list of appendices** here for all additional materials submitted (e.g., Appendix A – Informed Consent; Appendix B – Interview Guide, etc.):

Appendix A – Consent Form(Main study)
Appendix B – PAR-Q Questionnaire
Appendix C – Survey sheet for training log
Appendix D – Food Record Form
Appendix E – Food record Instructions
Appendix F – Study Protocol
Appendix G – Flyer
Appendix. H – Sponsored research collaboration agreement
Appendix. I – Consent form (pilot study)
Appendix. J – VO2Max test protocol

13. PARTICIPANTS AND/OR DATA

(a) Describe the participants to be recruited, or the individuals about whom personally identifiable information will be collected. List the inclusion and exclusion criteria. Where the research involves extraction or collection of personally identifiable information, please describe from whom the information will be obtained, what it will include, and how permission to access the data is being sought. (Strategies for recruitment are to be described in section #15.) Where applicable, justify the sample size.

In consultation with a biostatistician at the Hospital for Sickkids, sample size calculation with the Indicator Amino Acid Oxidation (IAAO) technique is exceedingly difficult by traditional statistical approaches. As such, we are ultimately limited to previously experiences with this technique to guide the powering of the present study. The technique works by establishing a breakpoint after modelling the collective data (i.e. all metabolic trials) as a single cohort with a bi-phase linear regression approach; as such, a breakpoint could ultimately be delineated with as few as 2-3 participants (i.e. 14-21 metabolic trials) but with the major drawback being an exceedingly large confidence interval (due to the likelihood of inter-individual response variations). Therefore, traditionally these trials have been designed to test the minimum number of participants (i.e. total metabolic trials) that will provide a reasonably narrow confidence interval (CI) (from which population requirements can be estimated) for the breakpoint. As there is a diminishing return on the narrowing of the CI with greater numbers of participants tested, past experience with this technique has revealed a similar CI with 5-12 sedentary participants (35-82 metabolic trials). For example, below is a brief list of studies utilizing the IAAO technique and their total metabolic trials and associated CI.

Humayan et al., 2007. 8 participants for 56 total metabolic trials: CI of ~33% for the estimated average requirement (i.e. breakpoint).

Elango et al., 2011. 8 participants for a 56 total metabolic trials: CI of ~20% for the estimated average requirement (i.e. breakpoint). Model prediction of r^2 = 0.75.

Kriengsinyos et al., 2004: 5 participants for a total of 35 metabolic trials: CI ranging from 17-35% for the estimated average requirement (i.e. breakpoint).

Tang et al., 2014.: 6 participants for a total of 42 metabolic trials: CI ~35% for the estimated average requirement (i.e. breakpoint).

Rafii et al., 2015.: 12 participants for a total of 82 metabolic trials: CI of 34% for the estimated average requirement (i.e. breakpoint).

As this study is the first to utilize the IAAO in trained individuals after a bout of endurance exercise, we have targeted 8 participants to increase our likelihood of optimizing the CI. In addition, utilization of 8 participants (56 total metabolic trials) has achieved excellent model predictions (i.e. $r^2 = 0.75$) and CI (~20%) when determining protein requirements in children utilizing the same methodology (Elango R et al., 2007).Therefore, we will recruit 8 participants in the present study, which is a target recruitment level that is in line with our previously approved study examining protein requirements in physically active young adults after a bout of variable intensity exercise (Title: “Amino acid metabolism and basic requirements in active, young adults”; REB #: 29654).

We will recruit 8 participants for the main study for a target of 56 total metabolic trials (i.e. 8 participants at 7 different protein levels). Although participant dropout has been extremely limited in previous studies utilizing this methodology in sedentary populations (personal communication, Prof. Paul Pencharz, Hospital for Sickkids), we have targeted this recruitment level to account for a potential 25% drop out rate, which would still provide us 6 participants (42 metabolic trials) and allow us to accurately determine the estimated average protein requirement with this methodology. Nevertheless, we believe that our screening during the introductory session prior to participants signing the consent form (and hence being officially enrolled in the study) will allow us to fully inform the participants and to subsequently enroll highly motivated individuals, thereby minimizing any potential issues with participant drop out and/or inability to perform the exercise task.

In pilot study, we will recruit 4 participants which is a target recruitment level that is in line with our previously approved study examining protein requirements in young healthy adults (Bross R et al., 1998)

Inclusion Criteria :

- Healthy, male, endurance-trained participants who regularly more than 40 km/week
- Ability to perform the exercise stimulus (20 km run) on metabolic trial.
- Subjects will be 18-35 years old.

Exclusion Criteria:

- Inability to meet health and physical activity guidelines according to the PAR-Q+ (The Physical Activity Readiness Questionnaire for everyone; Appendix. B).
-
- Female: hormonal fluctuations associated with the menstrual cycle have been reported to alter protein metabolism during exercise (Moore, Camera, Areta, and Hawley, 2014; Hamadeh, Devries, and Tarnopolsky 2005) and may influence specific amino acid requirements at rest (Kriengsinyos, Wykes, Goonewardene, Ball, and Pencharz 2004); as such, the present study will test the effect of endurance exercise on protein requirements in males only to ensure a stable hormonal environment and to increase the homogeneity of the physiological response. Nevertheless, given that WHO/FAO protein requirements are provided independently of sex and that recommendations for protein intake in athletes generally do not distinguish between the sexes (American Dietetic A, Dietitians of C, American College of Sports, Rodriguez, Di Marco, and Langley, 2009) or that protein requirements have been reported to be lower in endurance trained females than males (Rowland and Wadswortt, 2011), it could also be argued that protein requirements determined by IAAO would be similar between the sexes and/or potential reduced in females. Therefore, to investigate the effect of endurance exercise on protein requirements, we will test males as an indication of the potentially greatest protein requirement between the sexes.
- Inability to adhere to any of the protocol guidelines (i.e. alcohol, caffeine consumption)
- Regular tobacco use (screened by survey sheet for training log (Appendix.C))
- Illicit drug use (e.g. growth hormone, testosterone, etc.) (screened by survey sheet for training log (Appendix. C))

REFERENCES

American Dietetic A, Dietitians of C, American College of Sports M, Rodriguez NR, Di Marco NM, and Langley S. American College of Sports Medicine position stand. Nutrition and athletic performance. Medicine and science in sports and exercise 41: 709-731, 2009.

Bross R, Ball RO, Pencharz PB. Development of a Minimally Invasive Protocol for the Determination of Phenylalanine and Lysine Kinetics in Humans during the Fed State. J. Nutr. 128: 1913–1919, 1998.

Elango R, Humayun MA, Ball RO, and Pencharz PB. Lysine requirement of healthy school-age children determined by the indicator amino acid oxidation method. Am J Clin Nutr 86(2): 360-365, 2007.

Elango R, Humayun MA, Ball RO, and Pencharz PB. Protein requirement of healthy school-age children determined by the indicator amino acid oxidation method. Am J Clin Nutr 94: 1545-1552, 2011.

Hamadeh MJ, Devries MC, and Tarnopolsky MA. Estrogen supplementation reduces whole body leucine and carbohydrate oxidation and increases lipid oxidation in men during endurance exercise. The Journal of clinical endocrinology and metabolism 90: 3592-3599, 2005.

Humayun MA, Elango R, Ball RO and Pencharz PB. Reevaluation of the protein requirement in young men with the indicator amino acid oxidation technique. Am J Clin Nutr 86: 995-1002, 2007.

Kriengsinyos W, Wykes LJ, Goonewardene LA, Ball RO, and Pencharz PB. Phase of menstrual cycle affects lysine requirement in healthy women. American journal of physiology Endocrinology and metabolism 287: E489-496, 2004.

Moore DR, Camera DM, Areta JL, Hawley JA. Beyond muscle hypertrophy: why dietary protein is important for endurance athletes. Appl Physiol Nutr Metab. 2014 Sep;39(9):987-97. doi: 10.1139/apnm-2013-0591. Epub 2014 Feb 7.

Pillai RR, Elango R, Muthayya S, Ball RO, Kurpad AV, Pencharz PB. Lysine requirement of healthy, school-aged Indian children determined by the indicator amino acid oxidation technique. J Nutr 140(1):54-59, 2009.

Rafii M, et al., 2015. Dietary protein requirement of female adults >65 years determined by the indicator amino acid oxidation technique is higher than current recommendations. J. Nutr. 145:18-24.

Roberts SA, Thorpe JM, Ball RO, and Pencharz PB. Tyrosine requirement of healthy men receiving a fixed phenylalanine intake determined by using indicator amino acid oxidation. Am J Clin Nutr (73(2):276-282. 2001.

Tang M, et al. 2014. Assessment of protein requirement in octogenarian women with use of the indicator amino acid oxidation technique. Am. J. Clin. Nutr. 99:891-8.

Rowlands DS and Wadsworth DP. Effect of high-protein feeding on performance and nitrogen balance in female cyclists. Medicine and science in sports and exercise 43: 44-53, 2011.

(b) Is there any group or individual-level vulnerability related to the research that needs to be mitigated (for example, difficulties understanding informed consent, history of exploitation by researchers, power differential between the researcher and the potential participant)?

There exists no group, or individual-level vulnerability related to the research that needs to be mitigated.

14. EXPERIENCE OF INVESTIGATORS WITH THIS TYPE OF RESEARCH

(a) Please provide a brief description of previous experience with this type of research by (i) the principal investigator/supervisor or sponsor, (ii) the research team and (iii) the people who will have direct contact with the participants. If there has not been previous experience, please describe how the principal investigator/research team will be prepared.

The principal investigator (Dr. Dan Moore) has expertise on the effect of exercise on amino acid metabolism in adults and has conducted similar research on the protein requirements of active adults using by IAAO technique.<br>Visiting researcher, Hiroyuki KATO has experienced in the design and implementation of exercise and nutritional intervention in elderly people, and set this protocol with PI. He also has comprehensive knowledge of the study design and its potential impact on the participants.

(b) For projects that will involve community members (e.g., peer researchers) in the collection and/or analysis of data, please describe their status within the research team (e.g., are they considered employees, volunteers or participants?) and what kind of training they will receive?

Peer researchers (students, volunteers) will be required to obtain the most up-to-date Emergency Response Training from the University of Toronto in order to assist with the study. At all times throughout the testing protocol, at least one individual assisting the participants will have Standard First Aid training. Additionally, any peer researchers assisting with the study in any capacity will be required to have a strong understanding of the study protocol.

15. RECRUITMENT OF PARTICIPANTS

- Where there is recruitment, please describe how, by whom, and from where the participants will be recruited
- Where participant observation is to be used, please explain the form of insertion of the researcher into the research setting (e.g., living in a community, visiting on a bi-weekly basis, attending organized functions)
- If relevant, describe any translation of recruitment materials, how this will occur and whether or not those people responsible for recruitment will speak the language of the participants.
- **Attach a copy of all posters, advertisements, flyers, letters, e-mail text, or telephone scripts to be used for recruitment.**

Participants will be recruited from the student population at the University of Toronto or the member of the regional running club through the use of notices and flyer postings, and word-of-mouth.

16. COMPENSATION

Please see U of T's Compensation and Reimbursement Guidelines.

(a) Will participants receive compensation for participation?

Financial	Yes ☒	No ☐
In-kind	Yes ☐	No ☐
Other	Yes ☒	No ☐

(b) If **Yes**, please provide details and justification for the amount or the value of the compensation offered.

Proposed compensation is meant to adequately reimburse participants for any costs incurred, and to provide a token gift of appreciation for their effort. Compensation will be provided in the following rates: \$30/session2, and\$100/study day at the lab. This will cover parking (\$15/day), a small meal for the participant at the end of the day (~\$10), and a nominal gift of ~\$75 for adhering to the 2-d controlled diet and for the participant's effort.<br>Total value is \$730.

(c) If **No**, please explain why compensation is not possible or appropriate.

N/A

(d) Where there is a withdrawal clause in the research procedure, if participants choose to withdraw, how will compensation be affected?

Participants will be compensated based on the number of trials they complete, which is independent of if and when they withdraw their consent.

SECTION C –DESCRIPTION OF THE RISKS AND BENEFITS OF THE PROPOSED RESEARCH

17. POSSIBLE RISKS

(a) Please indicate all potential risks to participants as individuals or as members of a community that may arise from this research:

(i) Physical risks (e.g., any bodily contact or administration of any substance): Yes ☒ No ☐

(ii) Psychological/emotional risks (e.g., feeling uncomfortable, embarrassed, or upset): Yes ☒ No ☐

(iii) Social risks (e.g., loss of status, privacy and/or reputation): Yes ☐ No ☒

(iv) Legal risks (e.g., apprehension or arrest, subpoena): Yes ☐ No ☒

(b) Please briefly describe each of the risks noted above and outline the steps that will be taken to manage and/or minimize them.

All participants will be required to provide written informed consent in order to partake in the study. All risks inherent to the study will be presented in the written consent forms. All participants will have average or above average reading and comprehension abilities. Participation is voluntary, and there will be no pressure or time constraints regarding the decision to participate. There are no benefits to the subjects except for the monetary reward or compensation, as well as the good will of helping the progress of science research.

The research project involves an initial maximal exercise intervention that involves the participants to reach volitional fatigue. However, the research project will enroll young trained adults aged 18-35 who will be regularly engaged in high intensity endurance training. All participants will be screened for their health risks by a PAR-Q+ risk assessment form. The PAR-Q+ will be completed by the participants will suffice as a measure of the participant's ability to partake in the study. Any participants that do not meet the health requirements to partake in any of the physical tests (This treadmill-based fitness test, 20 km run) will be dropped from the study. Nevertheless, the metabolic risk of performing a maximal test or to complete the 20-km treadmill run during the trial in the healthy, endurance-trained population is minute (i.e. less than 1 in 10000) (Goodman et al., 2011).

There exists minimal risk to the participants as a function of the utilization of stable isotope kinetics in the study design. The non-invasive stable isotope model will administer a stable isotope orally with only minimal urine collection. The stable isotope ([^{13}C]phenylalanine) used in this study is naturally occurring (~1.1% of all carbon atoms are ^{13}C) and is metabolised within the body in exactly the same manner as 'normal' [^{12}C]phenylalanine. The IAOO technique has been utilized in populations ranging from neonates and children to pregnant women and the elderly, and has been specifically designed with minimal risk in mind. Additionally, the diet for each day will be specifically tailored to each individual's energy requirements and will provide 1.5X their resting energy expenditure. The exposure to the lower protein intake is minimized by use of the IAAO technique, which only requires them to consume this diet for 8h in comparison to the 7-10d using the traditional nitrogen balance technique. Participants will be provided with a diet that will meet their energy and macronutrient and micronutrient needs and therefore poses no risk to their health.

During the maximal fitness test, participants will exercise until volitional fatigue on the treadmill. Therefore, there is a theoretical risk that participants may fall on the treadmill upon completion of the test. However, this risk will be minimized by familiarizing the participants with running on the treadmill and using the handrails provided to lift themselves off of the treadmill upon completion of the test. In addition, participants will be closely monitored during the entire time while on the treadmill (including a "spotter" at the end of the treadmill) to ensure the risk of falling is minimized.

There is little physical risk to the participants performing the 20-km treadmill run on the metabolic trial days as participants will be regularly active (i.e. >40km of training/week) and will generally be familiar with long training runs. In addition, the exercise will be performed at a self-selected pace and therefore will be individually tailored to each participants comfort level and subsequently be similar to their habitual training.

As this is a 7-arm crossover trial with a 2-day controlled diet and 10-h study day per trial, there may be some inconvenience with the number of days required to complete the study and the travel to the university. However, we will accommodate the participant's schedule when arranging the trials to ensure it minimally disrupts their habitual training and general lifestyle.
The investigational beverages to be consumed during the metabolic trials will use crystalline amino acids, which can have an unpleasant taste to some individuals. To minimize this discomfort, the drinks will be flavoured with additional carbohydrate and artificial sweeteners to improve palatability. In addition, protein-free cookies will be used to supplement the liquid diet and will be provided after each hourly drink, which will help cleanse the palate and reduce any aftertaste incurred by the drinks.

During the body composition analysis in the Bodpod, individuals who are uncomfortable with small enclosed spaces may experience some anxiety. We will ensure participants are informed of the Bodpod procedure and will be provided with an opportunity to see the equipment and sit in it (if they choose) ahead of time to gauge their comfort level, if they self-report a hesitation with the test. Nevertheless, the Bodpod test has features that would be conducive to alleviating any claustrophobic tendancies such as: i) inclusion of a cut-out window in the unit, which will allow participants to see out of the Bodpod and the investigators to see in (to assess the potential level of facial anxiety); ii) the Bodpod test can be easily cancelled by the participant at any time by pressing an illuminated blue button on the seat inside the unit, and; iii) the test duration is a quick as 3-5min scan.

Reference: Goodman JM, Thomas SG, Burr J. (2011). Evidence-based risk assessment and recommendations for exercise testing and physical activity clearance in apparently healthy individuals. Appl. Physiol. Nutr. Metab. 36:S14-32.

18. POSSIBLE BENEFITS

- Describe any potential direct benefits to participants from their involvement in the project
- Describe any potential direct benefits to the community (e.g., capacity building)
- Comment on the potential benefits to the scientific/scholarly community or society that would justify involvement of participants in this study

The participants will not directly benefit from participation in this study.

The community will not directly benefit from this research.

The scientific / scholarly community will directly benefit from this study. Recent research has suggested that current amino acid requirements may be underestimates of actual requirements. Additionally, the impact that physical activity has on amino acid requirements is not well understood in the demographic group being studied. Given global initiatives to increase physical activity levels, the information generated from this study will be invaluable to establish the basic nutritional requirements of active individuals, and will have far-reaching implications.

SECTION D – INFORMED CONSENT

19. CONSENT PROCESS

(a) Describe the process that will be used to obtain informed consent and explain how it will be recorded. Please note that it is the quality of the consent, not the form that is important. The goal is to ensure that potential participants understand to what they are consenting.

(b) If the research involves extraction or collection of personally identifiable information from or about a research participant, please describe how consent from the individuals or authorization from the data custodian (e.g., medical records department, district school board) will be obtained.

Participants will receive a copy of the consent and introduction documents prior to reporting to the lab. Written informed consent for the study in its entirety will be acquired at the end of the introductory session. Participants will first be verbally introduced to the entire process and protocol, ensuring that clear expectations have been outlined. This will include a detailed review of the study timeline, a demonstration of all measures to be used (ex – BodPod, HR monitors, accelerometers, treadmill tests where applicable, etc), and an oral assessment of any risks associated with the protocols. We will ensure that participants fully understand what the study involves by asking for feedback (for example – Do you understand?) throughout the detailed introduction to the study. Participants will then have the opportunity to ask questions, and careful time will be taken to address concerns. They will be given a thorough explanation, an opportunity to ask questions, and an opportunity to review the study in private prior to signing the consent form. The design of the study will also allow for repeated contact with the participants where additional questions can be answered and voluntary consent can be confirmed. Thus, a continuous consent process will be administered to ensure that participants are comfortable in partaking in all portions of the study. Please see the appendices for a copy of the consent form.

20 CONSENT DOCUMENTS

(a) **Attach a copy of the Information Letter/Consent Form.**
For details about the required elements in the information letter and consent form, please refer to our informed consent guide (**http://www.research.utoronto.ca/wp-content/uploads/2010/01/GUIDE-FOR-INFORMED-CONSENT-April-2010.pdf**)

Additional documentation regarding consent should be provided such as:

- **screening materials introductory letters, letters of administrative consent or authorization**

(b) If any of the information collected in the screening process - prior to full informed consent to participate in the study - is to be retained from those who are later excluded or refuse to participate in the study, please state how potential participants will be informed of this course of action and whether they will have the right to refuse to allow this information to be kept.

No collection of data prior to consent will occur.

21. COMMUNITY AND/OR ORGANIZATIONAL CONSENT, OR CONSENT BY AN AUTHORIZED PARTY

(a) If the research is taking place within a community or an organization which requires that formal consent be sought prior to the involvement of individual participants, describe how consent will be obtained and attach any relevant documentation. If consent will not be sought, please provide a justification and describe any alternative forms of consultation that may take place.

N/A.

(b) If any or all of the participants are children and/or others who are not competent to consent, describe the process by which capacity/competency will be assessed, and the proposed alternate source of consent.

i) Submit a copy of the permission/information letter to be provided to the person(s) providing the alternative consent

ii) Describe the assent process for participants and attach the assent letter.

N/A.

22. DEBRIEFING and DISSEMINATION

(a) If deception or intentional non-disclosure will be used in the study, provide justification. Please consult the Guidelines for the Use of Deception and Debriefing in Research

No deception of non-disclosure is being used.

(b) Please provide a copy of the written debriefing form, if applicable.

N/A.

(c) If participants and/or communities will be given the option of withdrawing their data following the debriefing, please describe this process.

In keeping with Good Clinical Practice Guidelines issued by the Food and Drug Administration (see: http://www.fda.gov/downloads/RegulatoryInformation/Guidances/UCM126489.pdf), any data collected and analysed prior to participant withdrawal will remain in the study database to maintain scientific validity and study power.

(d) Please describe what information/feedback will be provided to participants and/or communities after their participation in the project is complete (e.g., report, poster presentation, pamphlet, etc.) and note how participants will be able to access this information.

Participants will have the option of having their personal information (Age-Predicted VO_2-max, body composition) and the final results of the study sent to them. If they choose to receive this information, it will be e-mailed to them.

23. PARTICIPANT WITHDRAWAL

(a) Where applicable, please describe how participants will be informed of their right to withdraw from the project and outline the procedures that will be followed to allow them to exercise this right.

Participants will be told prior to providing informed consent that they may withdraw from the study at any time. There will be no formal procedure for withdrawal other than the participant expressing their intent to withdraw from the study, which may be done so in person or via e-mail. With the repeated nature of the study, there will

be ample opportunity for participants to confirm or remove their consent and subsequent participation in the study. The right to withdraw at any time is also outlined on the consent form.

(b) Indicate what will be done with the participant's data and any consequences which withdrawal may have on the participant.

The only consequence the participant will face if they choose to withdraw from the study pertains to compensation. Subjects will be compensated through the duration of their participation in the study on a per trial basis. If a participant chooses to withdraw from the study, any previous data that has been collected may still be used in the study analysis. In the event that a participant does withdraw from the study, any data collected and analysed prior to participant withdrawal will remain in the study database to maintain scientific validity and study power.

(c) If participants will not have the right to withdraw from the project at all, or beyond a certain point, please explain. Ensure this information is included in the consent process and consent form.

Participants may withdraw from the project at any point without penalty. In the event of voluntary withdrawal, the participant's reimbursement will be pro-rated at $100 per study day in the lab.

SECTION E – CONFIDENTIALITY AND PRIVACY

24. CONFIDENTIALITY

Data security measures must be consistent with UT's *Data Security Standards for Personally Identifiable and Other Confidential Data in Research*. All identifiable electronic data that is being kept outside of a secure server environment must be encrypted, consistent with the standards described at: *http://www.utoronto.ca/security/UTORprotect/encryption_guidelines.htm:*

(a) Will the data be treated as confidential? Yes ☒ No ☐

(b) Describe the procedures to be used to protect the confidentiality of participants or informants, where applicable

All personal information, including results of the many tests, will be kept strictly confidential. All results will be coded with a study identification number. The document connecting a participant's name to their identification number will be kept in a locked filing cabinet in the primary investigator's office. All study data inputted into a computer will be password protected, and access will be limited to study investigators only.

(c) Describe any limitations to protecting the confidentiality of participants whether due to the law, the methods used, or other reasons (e.g., a duty to report)

Not applicable.

25. DATA SECURITY, RETENTION AND ACCESS

(a) Describe how data (including written records, video/audio recordings, artifacts and questionnaires) will be protected during the conduct of the research and dissemination of results.

All data will be stored using a participant's coded number, within a locked cabinet located in the principle investigator's office.

(b) Explain how long data will be retained. (If applicable, referring to the standard data retention practice for your discipline) Provide details of their final disposal or storage. Provide a justification if you intend to store your data for an indefinite length of time. If the data may have archival value, discuss how participants will be informed of this possibility during the consent process.

All paper documentation pertaining to individual participants will be shredded five years following the study's completion. At this same time point, all electronic data pertaining to individual participants will also be destroyed. The means and standard deviation values for each group will be retained for potential future reference.

(c) If participant anonymity or confidentiality is not appropriate to this research project, please explain.

N/A.

(d) If data will be shared with other researchers or users, please describe how and where the data will be stored and any restrictions that will be made regarding access.

N/A.

SECTION F – LEVEL OF RISK AND REVIEW TYPE

See the *Instructions for Ethics Review Protocol Submission Form* for detailed information about the Risk Matrix.

26. RISK MATRIX: REVIEW TYPE BY GROUP VULNERABILITY and RESEARCH RISK

(a) Indicate the Risk Level for this project by checking the intersecting box

	Research Risk		
**Group Vulnerability**	**Low**	**Medium**	**High**
**Low**	**1** ☐	**1** ☒	**2** ☐
**Medium**	**1** ☐	**2** ☐	**3** ☐
**High**	**2** ☐	**3** ☐	**3** ☐

(b) Explain/justify the level of research risk and group vulnerability reported above:

Research Risk – The research project involves an initial maximal exercise intervention (treadmill test) that involves the participants to reach volitional fatigue. Also, the protocol will implement minimally invasive stable isotope kinetics. Although the utilization of isotope kinetics, and maximal exercise interventions have both been validated in previous work in the demographic groups participating the current study, the nature of the protocol corresponds to a research risk rating of medium. However, the non-invasive stable isotope model will administer a stable isotope orally with only minimal urine collection. This technique has been utilized in populations ranging from neonates and children to pregnant women and the elderly, and has been specifically designed with minimal risk in mind.

Group Vulnerability – The research project will enroll young adults aged 18-35. All participants will be healthy and of relatively high fitness levels. For this reason, the group vulnerability of this project corresponds to a rating of low.

(Please note that the final determination of Review Type and level of monitoring will be made by the reviewing University of Toronto REB)

Based on the level of risk, these are the types of review that a protocol may receive:

Risk level = 1: Delegated Review; Risk level = 2 or 3: Full Board Review

For both delegated and full reviews (SSH&E, HS, or HIV), please submit one electronic copy of your protocol and all appendices (e.g., recruitment, information/consent and debriefing materials, and study instruments) as a **single** Word document or a pdf. *Do not submit your entire research proposal.* Please ensure that the electronic signatures are in place and e-mail to **new.ethics.protocols@utoronto.ca**

The deadline for delegated review (SSH&E or HS) is EVERY Monday, or first business day of the week, by 4 pm. Information about full REB meeting and submission due dates are posted on our website (SSH&E, HS or HIV).

HIV REB reviews all protocols at full board level but applies proportionate review based on the level of risk.

All other submissions (e.g., amendments, adverse events, and continuing review submissions) should be sent to ethics.review@utoronto.ca

SECTION G – SIGNATURES

27. PRIVACY REGULATIONS

My signature as Principal Investigator, in Section G of this protocol form, confirms that I am aware of, understand, and will comply with all relevant laws governing the collection and use of personally identifiable information in research. I understand that for research involving extraction or collection of personally identifiable information, provincial, national and/or international laws may apply and that any apparent mishandling of personally identifiable information must be reported to the Office of Research Ethics.

For U of T **student researchers**, my signature confirms that I am a registered student in good standing with the University of Toronto. My project has been reviewed and approved by my advisory committee or equivalent (where applicable). If my status as a student changes, I will inform the Office of Research Ethics.

Signature of Investigator: ________________________________	Date:

For **Graduate Students**, the signature of the Faculty Supervisor is required. For **Post-Doctoral Fellows** and **Visiting Professors or Researchers**, the signature of the Faculty Sponsor is required. In addition to the supervisor/sponsor, the chair or the dean of the department is required to approve and sign the form

As the **Faculty Supervisor** of this project, my signature confirms that I have reviewed and approve the scientific merit of the research project and this ethics protocol submission. I will provide the necessary supervision to the student researcher throughout the project, to ensure that all procedures performed under the research project will be conducted in accordance with relevant University, provincial, national or international policies and regulations that govern research involving human subjects. This includes ensuring that the level of risk inherent to the project is managed by the level of research experience that the student has, combined with the extent of oversight that will be provided by the Faculty Supervisor and/or On-site Supervisor.

As the **Faculty Sponsor** for this project, my signature confirms that I have reviewed and approve of the research project and will assume responsibility, as the University representative, for this research project. I will ensure that all procedures performed under the project will be conducted in accordance with all relevant University, provincial, national or international policies and regulations that govern research involving human participants.

Signature of Faculty Supervisor/Sponsor: [signature] Date: August 26, 2014

As the **Departmental Chair/Dean**, my signature confirms that I am aware of the requirements for scholarly review and that the ethics protocol for this research has received appropriate review prior to submission.

In addition, my administrative unit will follow guidelines and procedures to ensure compliance with all relevant University, provincial, national or international policies and regulations that govern research involving human participants. My signature also reflects the willingness of the department, faculty or division to administer the research funds, if there are any, in accordance with University, regulatory agency and sponsor agency policies.

Print Name of Departmental Chair/Dean (or designate) :

Signature of Departmental Chair/Dean: ______________________________ Date:
(or designate)
