# Supplementary material for: Protein Requirements Are Elevated in Endurance Athletes after Exercise as Determined by the Indicator Amino Acid Oxidation Method
Source: PLoS One. 2016 Jun 20;11(6):e0157406. doi: 10.1371/journal.pone.0157406 (PMC4913918; doi:10.1371/journal.pone.0157406)
Supplement: S1 Text — (DOCX) [file pone.0157406.s003.docx]

**S1 text. The pilot study to determine an isotopic and metabolic steady state during metabolic trial.**

**Purpose**

The objective of this pilot study was to determine whether an isotopic steady state could be reached in breath ^13^CO_2_ enrichment without prior adaptation to the study meal. This was examined by determining the pattern and stability of background ^13^CO_2_ enrichment in breath and VCO_2_, while subjects consumed the experimental diet without containing L-[1-^13^C]phenylalanine.

**Methods**

Four subjects took part in this pilot study (**Supplemental** **Table 1**). The study design was based on the previous study (1) modified with an addition of the exercise stimulus (summarized in **Supplemental Figure 1**). The protocol was same as the protocol in our metabolic trial day except for the sample collection and the test diet. The test diets supplied 1.4 g protein/kg BW/day, 9.0 g Carbohydrate/kg BW/day and 1.6 * resting energy expenditure (REE) plus the exercise-induced energy expenditure (EEE) without substituting L-[1-^13^C]phenylalanine. Breath samples were collected every 30 min from 15 min to 465 min after ingesting the first hourly meal to determine ^13^CO_2_ enrichment (summarized in Supplemental Fig.1). VCO_2_ was measured over 20-min continuous intervals every 60 min from 460 min after ingesting the first hourly meal.

**Supplemental Table 1** Characteristics of participants

|  | Mean ± SD |
| --- | --- |
| Age, yr | 29.0 ± 5.0 |
| Height, cm | 174.5 ± 4.4 |
| Body weight, kg | 68.6 ± 9.1 |
| Fat-free mass, kg | 59.6 ± 8.5 |
| VO2peak, ml/kg/min | 59.5 ± 5 |
| REE, Kcal/day | 1745.1 ± 221.2 |

4 participants participated in this study.

REE, resting energy expenditure

**Supplemental Figure 1. The protocols employed for each metabolic trial.** 4 subjects performed metabolic trial once per subject.

**Statistical analysis**

The significance of the change in background enrichment of ^13^CO_2_ over the 8 h on the metabolic trial was tested by regression analysis (Graphpad Prism® 5; GraphPad Software, Inc.,La Jolla, CA). Establishment of isotopic steady state was evaluated by repeated linear regression analysis in which data points, beginning at time 0 min, were removed until a regression line with a slope not different from zero was achieved.

**Results**

The effect of hourly meals on background ^13^CO_2_ enrichment in breath and VCO_2_ after exercise stimuls was shown in **Supplemental Figure 2, 3**. The slope of the ^13^CO_2_ enrichment 285 min after consuming the first hourly meal was not significnatly different from zero (P > 0.05). VCO_2_ was kept constant after 20 km run until the end of the metabolic trial.

**Supplemental Figure 2 Effect of exercise and hourly meal on enrichment of ^13^CO_2_ in breath.**

A plateau in ^13^CO_2_ enrichment was achieved at 285 min .Slopes of the ^13^CO_2_ enrichment vs. time regression lines were not significantly different from zero 285 min after the completion of 20 km run ( P > 0.05). Values are means ± SEM, N = 4.

**Supplemental Figure 3 Effect of exercise and hourly meal on VCO_2_.**

Slopes of the VCO_2_ vs. time regression lines were not significantly different from zero. Values are means ± SEM, N = 4.

**References**

1. Bross R, Ball RO, Pencharz PB. Development of a minimally invasive protocol for the determination of phenylalanine and lysine kinetics in humans during the fed state. The Journal of nutrition. 1998;128(11):1913-9.
